
JOURNAL INFORMATION
==============================
NLM Title Abbreviation: Atlanta Med Surg J (1884)
NLM Journal ID: 101251623

Atlanta Medical and Surgical Journal (1884)

Publisher: Biomedical Journal Digitization Project

ARTICLE INFORMATION
==============================
PMCID: PMC9201843
PMID: 35829600
Article version: 1
Subjects: Correspondence

Local Application in Gout

Print publication date: 1899 Jan
Volume: 15
Volume Series: New Ser
Issue: 11
First page: 744
Last page: 744
License: This work is free of known copyright restrictions. For more information, please see PMC Back Issue Digitization.
License URL: https://creativecommons.org/publicdomain/mark/1.0/

==============================

There are several items in your JOURNAL I can heartily commend.

With your old physicians of thirty years ago I was once well acquainted. Ford, of Augusta; Hon. Dr. Miller, Fears, Shackleford, and many others, most of whom have joined the silent majority. Does my old friend and *chum*, Colonel Israel Putnam, still live? If so, remember me kindly to him when you see him. By the way, what think you of our proposed "Law to regulate the practice of medicine in Texas"? Too cumbersome, too wordy. One board of medical examiners to meet quarterly at the capitol would have been sufficient, I think. I doubt its passing the Legislature.

You are at liberty to use this letter or any part of it as you like. I write it in great bodily pain. I suppose that I have cancer of the rectum, growing out of hemorrhoidal tumors, from which I have suffered for near half a century.

Wishing you a pleasant Christmas, a happy new year, and success for your JOURNAL, I am,

C. S. REEVES, M.D.

[NOTE.—It gives us pleasure to publish this reminiscent letter of Dr. Reeves. We wish there were others of the "old school" who would do likewise and give the readers of THE JOURNAL some ideas from "ante-bellum days."]

---

### LOCAL APPLICATION IN GOUT.

℞ Potassii iodi, Liniment saponis ⎱ aa .......... ʒ iv.
Ol. cajaputi, Ol. cari ⎱ aa .......... ʒ ss.
Spiritus rect .......... q. s. ad. ℥ vii.
M. Sig. Apply on lint and cover with a protective.

---

**NOTICE.—This JOURNAL and THE INTERNATIONAL JOURNAL OF SURGERY one year for $2.00 cash.**
